# Increased Risk of Fracture after Traumatic Amputation: A Nationwide Retrospective Cohort Study

**DOI:** 10.3390/healthcare12131362

**Published:** 2024-07-08

**Authors:** Hyeonjin Cho, Junhee Park, Bongseong Kim, Kyungdo Han, Hea Lim Choi, Dong Wook Shin

**Affiliations:** 1 International Healthcare Center, Samsung Medical Center, Seoul 06351, Republic of Korea; 2 Department of Digital Health, SAIHST, Sungkyunkwan University, Seoul 06355, Republic of Korea; 3 Center for Cohort Studies, Total Healthcare Center, Kangbuk Samsung Hospital, Sungkyunkwan University School of Medicine, Seoul 03181, Republic of Korea; 4 Department of Medical Statistics, Catholic University of Korea, Seoul 06591, Republic of Korea; 5 Department of Statistics and Actuarial Science, Soongsil University, Seoul 06978, Republic of Korea; 6 Department of Family Medicine/Executive Healthcare Clinic, Severance Hospital, Yonsei University College of Medicine, Seoul 03722, Republic of Korea; 7 Department of Clinical Research Design and Evaluation, SAIHST, Sungkyunkwan University, Seoul 16419, Republic of Korea

**Keywords:** amputation, traumatic, amputees, fracture, cohort studies

## Abstract

The physiological changes and alterations in gait following amputation may increase the risk of fractures. However, there is insufficient research on fracture risk in amputees. Therefore, this study intended to analyze whether the risk of new fractures increases after traumatic amputations. This population-based, retrospective cohort study used data from the Korean National Health Insurance System database. The study included 19,586 participants who had undergone an amputation and 76,645 matched controls. The incidence of any fracture and site-specific fractures (vertebral, hip, and others) according to amputation site(s) and severity of disability due to amputation were evaluated using Cox proportional hazard regression analysis. During the mean follow-up of 4.2 years, amputees had a higher incidence rate (IR) of any fracture (adjusted HR [aHR] 1.47, 95% CI 1.36–1.60), vertebral fracture (aHR 1.63, 95% CI 1.44–1.85), hip fracture (aHR 1.85, 95% CI 1.39–2.46), and other fracture (aHR 1.34, 95% CI 1.20–1.49) compared to that of controls. In the presence of disability, the risks were further increased and were highest among amputees with severe disabilities. All fracture risks were higher in amputees than they were in controls, regardless of lower limb or upper limb amputation. This cohort study demonstrated that traumatic amputees experienced higher incidence of all fractures than did individuals without amputations, and this risk increases with severity of disability. This finding underscores the importance of early screening and lifestyle interventions to address fracture risk in traumatic amputees.

## 1. Introduction

Amputation is the removal or loss of a body part due to trauma or disease (peripheral vascular disease and/or diabetes mellitus, cancer, infection) [1]. After limb amputation, amputees might become vulnerable to new fracture. A previous cross-sectional study of limb amputees demonstrated that the bone mineral density of the residual lower limb decreases compared to the opposite, intact lower limb because the residual limb is unloaded during recovery; consequently, the remaining lower limb faces increased risk of osteoporosis and fragility fractures at the hip compared to the intact limb [2]. A prospective study involving individuals with unilateral lower limb amputations revealed that these amputees exhibited low confidence in their balance [3], well below the indicative cutoff point of need for intervention [4]. Amputation of both limbs led to a loss of proprioceptive sensory feedback from direct foot contact with the ground [5], altered postural sway [6], and an altered gait pattern. A prior study involving 435 individuals with unilateral lower limb amputations revealed that 49.2% of the participants reported a fear of falling, and 52.4% had experienced a fall within the past year [7]. This rate of falls exceeded the 28.7% among community-dwelling elderly (≥65 years) individuals from US statistics in 2014 [8].

Most previous studies that evaluated fracture risk in amputees used data that were limited to lower limb amputees, had a limited sample size and/or short observation time, or did not have a control group (Supplementary Table S1) [2,3,6,7,9,10,11,12]. Considering that the upper limbs play a major role in gait function [13,14], it is possible that the risk of fractures may increase after upper limb amputation as well. Studies focusing exclusively on traumatic amputees are limited. Typically, traumatic amputees have fewer underlying diseases that decrease bone density or impair gait function than do amputees with conditions such as peripheral artery disease or diabetes [6,15,16,17,18,19,20]. Focusing on traumatic amputees, therefore, allows better control of confounders (other than amputations) when assessing the risk of fracture.

Therefore, in this retrospective cohort study using long-term follow-up data from a nationwide health-claims database, we evaluated the risk of fracture among participants with various levels of traumatic amputation, which may increase fracture risk through different mechanisms, compared to that of a matched control group.

## 2. Materials and Methods

### 2.1. Data Source

This research was conducted using data provided by the Korean National Health Insurance Service (KNHIS). The KNHIS is the primary healthcare provider for 97% of the Korean population, extending medical aid to the remaining 3% of individuals with the lowest income [21]. The KNHIS provides a health screening program every two years to all Koreans aged 40 and above, as well as to all employed individuals, regardless of age [22]. In this program, the KNHIS gathers comprehensive data on anthropometric measurements (height, weight, blood pressure, etc.), health-related behaviors (smoking habits, alcohol consumption, etc.), and laboratory results (lipid profiles, blood glucose, etc.) [23,24]. In addition to the information gathered from the health-screening program, the KNHIS also compiles medical data for each individual, including the utilization of healthcare facilities and prescriptions, which are categorized using the International Classification of Disease, 10th revision (ICD-10) diagnosis codes.

### 2.2. Study Population

A total of 59,392 individuals who experienced amputations between 2010 and 2018 were identified. The definition criteria for amputation were set using the International Classification of Diseases, 10th revision (ICD-10) codes, which include the following categories: Z89 (acquired absence of limb), S48 (traumatic amputation of shoulder joint), S58 (traumatic amputation of forearm), S68 (traumatic amputation of wrist, finger), S78 (traumatic amputation of hip), S88 (traumatic amputation of knee), and S98 (traumatic amputation of ankle, toe). We initially included 27,832 participants who had undergone health screening within two years prior to the index date. Subsequently, we excluded individuals younger than 40 years (*n* = 3148) and those with a history of the following medical conditions: (1) diabetes with complications including ulcer of the lower limb (ICD-10 code: E10.6, E11.6, E12.6, E13.6, E14.6, L97 and E11.621), (2) thromboangiitis obliterans (ICD-10 code: I73.1), (3) arterial embolism and thrombosis (ICD-10 code: I74), and (4) fracture. Participants with missing information (*n* = 788) and those who experienced a new fracture within one year after the index date (*n* = 236) were also excluded. Finally, a total of 19,586 amputees was included in the study.

**Figure 1:**
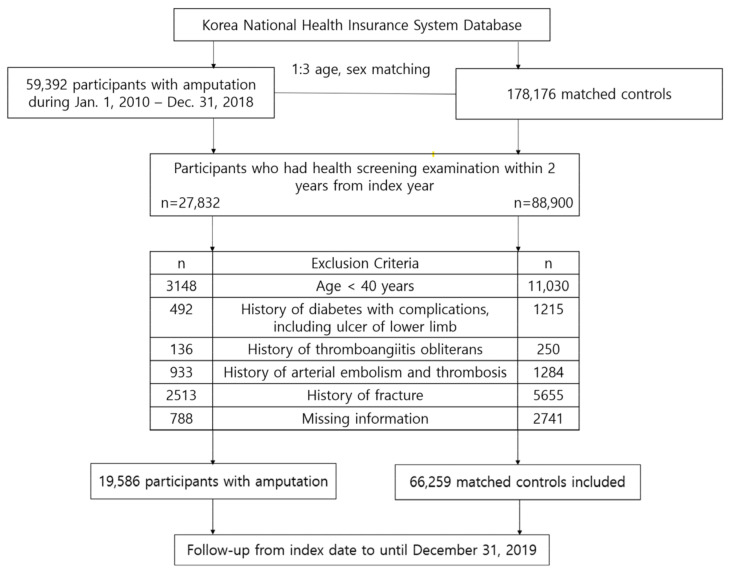
Study population selection flowchart.

We selected the control group using a 1:3 age- and sex-matching process, which was carried out sequentially each year. This technique ensured that participants who experienced amputation in a particular year were matched by age and sex with control participants who were alive in the same year. Matched control participants were assigned an index date corresponding to the date of amputation for their respective matched counterparts. The same exclusion criteria that were described were applied to the control subjects. The study population selection process is illustrated in Figure 1.

The Institutional Review Board of Samsung Medical Center approved this study (IRB File No. 2020-12-068). Written informed consent was not required by the review board because the data were publicly available and anonymized in accordance with confidentiality guidelines.

### 2.3. Severity of Disability due to Amputation

To evaluate the impact of disability, we included the disability grade documented in the National Disability Registry (NDR) managed by the Ministry of Health and Welfare [25]. In Korea, individuals with disabilities are registered to receive social benefits from the government. This registration process requires that a specialist physician diagnose the disability through a physical examination and x-ray assessment, and submission of verified documents. The degree of disability spans from grade 1, the most severe, to grade 6, the least severe (Supplementary Tables S2 and S3). Typically, amputation at proximal sites is associated with severe disability. For instance, a grade 1 disability due to lower limb amputation is defined as removal of both legs above the knee joint. In contrast, a grade 6 disability from a lower extremity amputation is defined as amputation of one leg above the tarsometatarsal joint. In the present study, individuals who were listed in the NDR within one year following the index date were categorized by disability. This group was further subdivided into those with mild disability (grades 4–6) and those with severe disability (grades 1–3).

### 2.4. Site of Amputation

To evaluate the specific association between amputation site and fracture, further analyses were performed considering the ICD codes for amputation sites. The upper limb was divided into shoulder/upper arm, forearm, and wrist/hand; the lower limb was divided into hip/thigh, lower leg, and ankle/foot.

### 2.5. Study Outcomes and Follow-Up

The study assessed newly diagnosed fractures as its main outcomes, including any fracture, vertebral fracture, hip fracture, and other fracture based on ICD-10 codes during follow-up. Vertebral fracture was identified by a claim using S22.0, S22.1, S32.0, M48.4, or M48.5 code at two or more outpatient visits [26,27,28,29]. Hip fracture was identified through a claim using S72.0 or S72.1 with one or more hospitalizations. Other fractures were identified by a claim indicating fractures of the upper arm (S42.0 fracture of clavicle; S42.2 fracture of upper end of humerus; S42.3 fracture of shaft of humerus), forearm (S52.5 fracture of lower end of radius; S52.6 fracture of lower end of ulna), or lower leg (S82.3 fracture of lower end of tibia; S82.5 fracture of medial malleolus; and S82.6 fracture of lateral malleolus) at two or more outpatient visits. Any fracture was identified through the occurrence of at least one or more fractures mentioned above. Observations for the cohort began at the index date and continued until the first instance of a fracture, death, or conclusion of the last follow-up on 31 December 2019.

### 2.6. Covariates

Age, sex, income level, and residential area data were obtained from the NHIS database for sociodemographic analysis. Alcohol consumption was categorized into non-drinker, mild to moderate (<30 g of alcohol/day) drinker, and heavy (≥30 g/day) drinker. Smoking status was categorized into never-smoker, former smoker, and current smoker. Regular physical activity was defined as moderate physical activity sessions lasting more than 30 min at least five times a week or vigorous physical activity sessions lasting more than 20 min at least three times week. Comorbidities were determined by analyzing both claims data prior to the screening date and health checkup findings. Hypertension was diagnosed by a claim of I10–I13 or I15, and use of antihypertensive medication or systolic/diastolic blood pressure ≥140/90 mmHg. Type 2 diabetes was diagnosed based on a claim with E11–E14 codes and use of antidiabetic medication or fasting glucose level ≥126 mg/dL. The definition of dyslipidemia included a claim with E78 codes and use of lipid-lowering medications or total cholesterol level ≥240 mg/dL. The assessment of overall comorbidity burden utilized the primary care equivalent of the Charlson comorbidity index (CCI) based on the ICD-10 code [30].

### 2.7. Statistical Analysis

A descriptive analysis was performed on the study subjects to compare the baseline characteristics of amputees to their matched controls. An independent *t*-test was utilized for continuous variables, while a chi-square test was applied to categorical variables. Cox proportional hazards regression was conducted to calculate the hazard ratio (HR) and 95% confidence interval (CI) for study outcomes in the amputation group. Each occurrence of a study outcome was independently followed and analyzed. Model 1 did not involve any adjustments for covariates. In Model 2, adjustments were made for age, sex, socioeconomic status (income level and place of residence), health-related behaviors (alcohol consumption, smoking, and regular physical activity), body mass index, and comorbidities (hypertension, type 2 diabetes, and dyslipidemia).

Statistical analyses were carried out using SAS version 9.4 (SAS Institute Inc., Cary, NC, USA). Statistical significance was defined as a *p*-value < 0.05.

### 2.8. Equity, Diversity, and Inclusion Statement

The authors conducted a study focusing on amputees, who have rarely been the topic of previous research, collecting data about amputation sites and the severity of disability due to amputation. Particularly, our research utilized KNHIS data encompassing the general older Korean population, including amputees with diverse genders, ages, and demographics. However, since our study was limited to Koreans, further research into other ethnicities is necessary.

## 3. Results

### 3.1. Baseline Characteristics

Table 1 provides an overview of the baseline characteristics of the study cohort. In the amputees, the mean age was 56.3 (standard deviation 9.5) years and 75.7% were male. Compared to controls, amputees showed higher likelihoods of rural residence, heavy drinking, current smoking, and were less likely to engage in physical activity. The amputees exhibited a higher prevalence of comorbidities like type 2 diabetes and dyslipidemia, and higher CCI scores than did the control participants.

### 3.2. Risk of Fracture among Amputees

**Figure 2:**
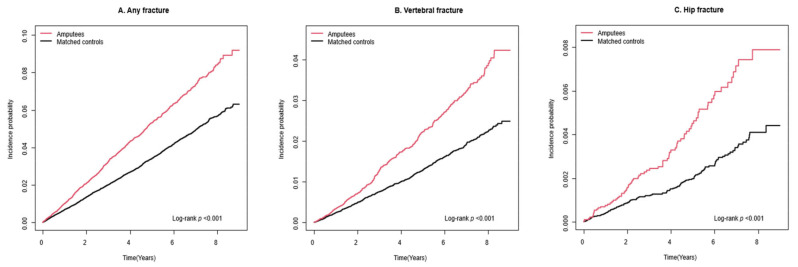
Incidence probability of fracture in participants with traumatic amputation and controls: (**A**) Any fracture, (**B**) Vertebral fracture, (**C**) Hip fracture.

**Figure 3:**
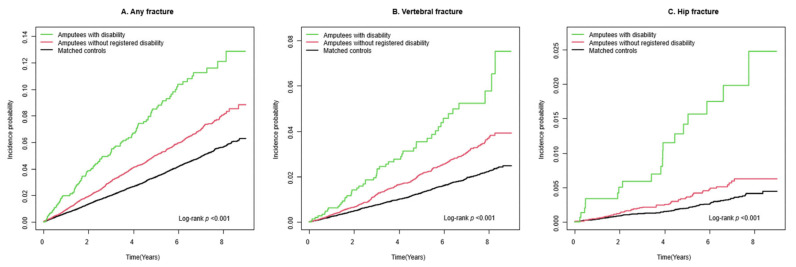
Incidence probability of fracture in participants with traumatic amputation with disability and without disability, and in controls: (**A**) Any fracture, (**B**) Vertebral fracture, (**C**) Hip fracture.

**Figure 4:**
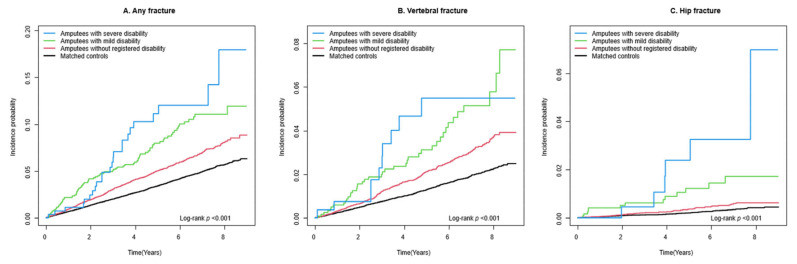
Incidence probability of fracture in participants with traumatic amputation without disability or with disability by severity degree and in controls: (**A**) Any fracture, (**B**) Vertebral fracture, (**C**) Hip fracture.

During a mean follow-up period of 4.2 (standard deviation 2.5) years, 883 incident cases of any fracture (10.8 per 1000 person-years), 374 incident cases of vertebral fracture (4.5 per 1000 person-years), 76 incident cases of hip fracture (0.9 per 1000 person-years), and 482 incident cases of other fracture (5.8 per 1000 person-years) were observed among the amputees (Table 2). Kaplan–Meier curves show that the incident probabilities of fractures in amputees were higher than they were in controls (log-rank *p* < 0.001) (Figure 2). Compared to controls, amputees had a higher risk of any fracture (adjusted HR [aHR] 1.47, 95% CI 1.36–1.60), vertebral fracture (aHR 1.63, 95% CI 1.44–1.85), hip fracture (aHR 1.85, 95% CI 1.39–2.46), and other fracture (aHR 1.34, 95% CI 1.20–1.49). The risks were further increased by the presence of disability (Figure 3): any fracture (aHR 2.07, 95% CI 1.71–2.50), vertebral fracture (aHR 2.16, 95% CI 1.63–2.88), hip fracture (aHR 3.98, 95% CI 2.45–6.47), and other fracture (aHR 1.70, 95% CI 1.29–2.25). Furthermore, those with severe disability had the highest risk of any fracture (aHR 2.59, 95% CI 1.73–3.87), vertebral fracture (aHR 2.51, 95% CI 1.34–4.69), hip fracture (aHR 6.73, 95% CI 2.95–15.35), and other fracture (aHR 1.91, 95% CI 1.02–3.56) (Figure 4).

### 3.3. Risk of Fracture According to Site of Amputation

Compared to controls, participants with forearm amputation were at higher risk of any fracture (aHR 2.68, 95% CI 1.28–5.63) and other fracture (aHR 3.36, 95% CI 1.40–8.10). Compared to controls, participants with wrist/hand amputation had a higher risk of any fracture (aHR 1.46, 95% CI 1.34–1.58), vertebral fracture (aHR 1.62, 95% CI 1.42–1.83), hip fracture (aHR 1.68, 95% CI 1.24–2.26), and other fracture (aHR 1.33, 95% CI 1.19–1.48). Compared to controls, participants with every type of lower limb amputation were at higher risk of hip fracture, including hip/thigh amputation (aHR 9.47, 95% CI 1.31–68.43), lower leg amputation (aHR 9.24, 95% CI 2.92–29.18), and ankle/foot amputation (aHR 4.71, 95% CI 1.92–11.54). Participants with ankle/foot amputation also had a higher risk of any fracture (aHR 1.89, 95% CI 1.25–2.85) and vertebral fracture (aHR 2.02, 95% CI 1.08–3.76) than did controls.

## 4. Discussion

This study demonstrated the increased risk of fractures among individuals with traumatic amputation. Our results align with previous findings of decreased bone mineral density, impaired gait balance, and increased incidence of falls among amputees (Supplementary Table S1) [2,3,7]. The fracture risk further increased with severity of disabilities. Individuals with hand or wrist amputations showed increased risk of various fractures, including vertebral and hip fractures. Those with below-hip amputation had an elevated risk of hip fractures.

Previous studies on newly occurred fractures in amputees primarily focused on the health outcomes caused by fractures or treatment methods for the fractures [12,31,32], rather than assessing the risk of new fracture incidence. Therefore, to the best of our knowledge, this is the first large, nationwide, population-based cohort study to evaluate fracture risk among traumatic amputees with disabilities. Our results reveal that amputees, in general, are at higher risk of fractures than are non-amputees. By exclusively including individuals with amputations resulting from traumatic injuries, we could assess the increased risk of fractures due to amputation alone, while eliminating the influence of other factors such as peripheral artery disease or diabetes (which might also increase the risk of fracture independently).

The higher risk of fractures in amputees, particularly in those with proximal amputation, can be explained through the frequent experience of phantom limb symptoms [33,34,35]. This phenomenon causes amputees to mistakenly perceive the presence of their amputated limb and can mislead them to bear weight on the side affected by amputation, increasing the risk of falls and related injuries. The closer the amputation site is to the proximal end of the limb, the longer it takes for the amputated distal portion to touch the ground and arrest the fall, leading to greater instability in posture and balance. As a result, individuals with proximal amputations may experience a higher physical force when impacting the ground in a fall related to the phantom limb sensation, which increases the likelihood of fractures.

In our study, compared to an amputee without registered disability, an amputee with disability had an increased risk of all types of fractures; this risk increased with disability severity. A systemic review of 25 studies on various levels of foot amputation reported a tendency for worsening gait function as the level of foot amputation progressed from distal to proximal. More proximal amputations caused abnormal gait, significant loss of power at the ankle joint, and hindered mobility [11]. As the level of amputation progresses from distal to proximal regions, more severe disability can result (Supplementary Tables S2 and S3). Therefore, it is reasonable to infer that an amputee with severe disability will experience a more pronounced decline in gait function, leading to an increased risk of falls.

As the level of amputation moves proximally toward the body center, physical activity declines, as does gait function [20]. A decrease in physical activity can lower overall bone density [36]. A study of lower limb amputees found that, compared to transtibial amputees, transfemoral amputees experienced a greater decrease in overall bone density [2,37,38]. Consequently, individuals with severe disabilities may be at a higher risk of fractures than those with minor disabilities because their reduced physical activity leads to a decline in bone density.

Amputees develop decreased overall bone density, including that in the residual lower limb compared to that in the unamputated lower limb [37,39,40,41,42]. Weight-bearing on the residual lower limb is reduced, which hinders the normal bone turnover process [43]. Therefore, the residual lower limb becomes vulnerable to fractures. If an individual experiences physical trauma or falls onto the residual lower limb, fractures can occur more easily than they might have prior to amputation. This increased vulnerability could elevate the risk of hip and other fractures, including lower limb fractures, in the residual limb.

Individuals with proximal amputations experience decreased mobility and are at risk of accidents when using facilities such as wheelchairs, elevators, stairs, and public transportation. A previous cross-sectional study reported that the hip area showed the highest prevalence of fractures due to frequent accidents during wheelchair transfer [44]. This finding mirrors our result, which indicated a particularly substantial increase in the risk of hip fracture compared to vertebral or other fractures in amputees with severe disability. Amputees with severe disability often experience amputation at a proximal portion of the limb and are more likely to depend on a wheelchair than are those without severe disability. Furthermore, wheelchair users are at risk of accidents involving elevator doors or traffic when crossing the road. Amputees with proximal amputation who do not depend on a wheelchair tend to walk more slowly than do those with distal amputation, and have similar difficulties to those mentioned above. Consequently, individuals with proximal amputation are susceptible to accidents that can increase their fracture risk. Overall, there is a need for further research to investigate the specific mechanisms and environments through which individuals with proximal amputations experience fracture. Efforts should be made to improve the factors that increase amputees’ risk of fractures.

In our study, significantly increased risk of fracture was found among participants with upper limb amputations (or forearm/wrist/hand amputations). This result might be due to an increase in imbalance risk and fall risk among those with upper limb amputation. It is not only the lower limbs that support body weight, the upper limbs also contribute significantly to balance in the human body through arm swing to offset leg-generated angular momentum and reduce overall body angular momentum [13]. A cross-sectional study of 105 participants with upper limb amputation at or proximal to the wrist found that 45.7% experienced at least one fall and 28.6% experienced two or more falls in the preceding year [45]. Wearing an upper limb prosthesis to facilitate daily activities increased the risk of fall by 5.8 fold [45] because the additional body mass can increase postural control demand [14]. When a person wearing an upper limb prosthesis falls, attempting to arrest the fall by reaching for a wall or the floor or grabbing onto a fixed object with an unfamiliar prosthesis can fail and potentially cause further trauma [45]. There is less attention to rehabilitation training that focuses on balance in patients with upper limb amputation [46] than there is in those with lower limb amputation [47]. Given the risks of falls and fractures after upper limb amputation, more attention must be paid to balance-targeted rehabilitation in such patients.

According to a previous study of 2461 patients with hand injuries, 28.8% of total amputations and protrusions occurred in the workplace [48]. Of these cases, 27.5% of the total causes of amputations and protrusions were attributed to machinery and tools [48]. According to the 2019 statistics on accident and injury victims in South Korea, 43% of a total of 10,734 amputations, cuts, and puncture cases occurred in the service industry. Specifically, 48.2% of those cases involved the food and accommodation sector. A substantial portion of these injuries involved the hands and occurred as a result of food processing equipment such as kitchen knives, meat cleavers, and vegetable slicers [49]. If individuals who suffered hand amputations continue to work in the same occupational environment, there is a continued risk of secondary fractures resulting from accidents with similar mechanisms. Since covariates related to the working environment were not adjusted in our study, it is possible that this working environment acted as a confounder and increased the risk of fractures. Therefore, it is important to devise methods to ensure compliance with safety rules for accident prevention within the workplace.

## 5. Clinical Implications

Amputees have an elevated likelihood of fractures as a result of impaired gait function, decreased bone density, and the phantom limb phenomenon following amputation. When amputation occurs close to the proximal end of the limb and is accompanied by severe disability, the consequences can be exacerbated, further increasing the risk of fractures. Even upper limb amputations can affect gait and balance and increase the risk of fractures. Furthermore, individuals who have experienced upper limb amputation during work may be at risk of recurring accidents in a similar work environment, which increases their susceptibility to fractures. Therefore, there is a clinical necessity to develop rehabilitation programs after amputation to encourage lifestyle changes (as necessary) and prevent subsequent fractures. Additionally, efforts should be made to improve work environments to prevent secondary occupational hazards when upper limb amputations occur in work settings.

## 6. Limitations

This study has several limitations. First, because our analysis relied on administrative data; specific clinical data, such as the mechanism and detailed cause of fractures, were not available. Second, amputees might have more frequent hospital visits compared to control subjects; therefore, a diagnosis of fracture may have been more likely in amputees than in controls, representing detection bias. Last, since the data used in our study were collected from participants in the KNHIS, there are limitations in terms of their generalizability.

## 7. Conclusions

This study identified an increased incidence of fractures in traumatic amputees compared to control subjects. The risk escalated with increasing levels of severity of disability increases. Screening and early lifestyle interventions for fractures are needed for amputees.

## Figures and Tables

**Table 1:** Baseline characteristics of the study population.

Variables	Matched Controls (*n* = 66,259)	Amputees (*n* = 19,586)	*p* Value
Age (years)	55.9 ± 9.5	56.3 ± 9.5	<0.001
Sex (male)	51,470 (77.7)	14,818 (75.7)	<0.001
Income (lowest 20%)	11,875 (17.9)	3579 (18.3)	0.261
Place of residence (urban)	30,447 (46.0)	6755 (34.5)	<0.001
Alcohol consumption			<0.001
None	29,967 (45.2)	9088 (46.4)	
Mild to moderate	29,508 (44.5)	7958 (40.6)	
Heavy	6784 (10.2)	2540 (13.0)	
Smoking status			<0.001
Never	29,517 (44.6)	8862 (45.3)	
Former	17,816 (26.9)	4523 (23.1)	
Current	18,926 (28.6)	6201 (31.7)	
Regular physical activity	15,284 (23.1)	3537 (18.1)	<0.001
Body mass index (kg/m^2^)	24.2 ± 3.0	24.1 ± 3.0	<0.001
Systolic blood pressure (mmHg)	124.6 ± 14.4	124.6 ± 14.9	0.849
Diastolic blood pressure (mmHg)	77.7 ± 9.9	77.6± 10.0	0.197
Fasting glucose (mg/dL)	102.4 ± 25.4	102.9 ± 27.7	0.020
Total cholesterol (mg/dL)	197.6 ± 37.6	196.5 ± 37.6	<0.001
Comorbidities			
Hypertension	24,129 (36.4)	7267 (37.1)	0.080
Type 2 diabetes	8953 (13.5)	2763 (14.1)	0.033
Dyslipidemia	19,016 (28.7)	5447 (27.8)	0.016
Charlson comorbidity index	1.3 ± 1.6	1.6 ± 1.7	<0.001

Data are presented as number (%) or mean ± standard deviation.

**Table 2:** Hazard ratios and 95% confidence intervals for incidence of fractures among amputees compared to matched controls.

	N	Any Fracture			Vertebral Fracture			Hip Fracture			Other Fracture		
		Cases/IR	Model 1	Model 2	Cases/IR	Model 1	Model 2	Cases/IR	Model 1	Model 2	Cases/IR	Model 1	Model 2
Matched controls	66,259	2014/7.1	1 (ref.)	1 (ref.)	772/2.7	1 (Ref.)	1 (Ref.)	132/0.5	1 (Ref.)	1 (Ref.)	1,201/4.2	1 (Ref.)	1 (Ref.)
Amputees	19,586	883/10.8	1.54 (1.42–1.66)	1.47 (1.36–1.60)	374/4.5	1.69 (1.49–1.91)	1.63 (1.44–1.85)	76/0.9	2.00 (1.51–2.65)	1.85 (1.39–2.46)	482/5.8	1.40 (1.26–1.55)	1.34 (1.20–1.49)
**Presence of disability**													
No disability	18,061	769/10.3	1.45 (1.34–1.58)	1.41 (1.30–1.54)	323/4.2	1.59 (1.39–1.81)	1.57 (1.38–1.79)	57/0.7	1.63 (1.20–2.23)	1.57 (1.14–2.15)	42/7.5	1.36 (1.21–1.51)	1.30 (1.17–1.45)
Disability	1525	114/17.4	2.45 (2.03–2.96)	2.07 (1.71–2.50)	51/7.5	2.80 (2.11–3.72)	2.16 (1.63–2.88)	19/2.8	6.05 (3.74–9.79)	3.98 (2.45–6.47)	10/8.5	1.84 (1.40–2.43)	1.70 (1.29–2.25)
**Severity of disability**													
Mild	1249	90/16.6	2.34 (1.90–2.89)	1.97 (1.59–2.43)	41/7.3	2.72 (1.99–3.73)	2.09 (1.53–2.87)	13/2.3	5.00 (2.83–8.85)	3.36 (1.89–5.95)	42/7.5	1.80 (1.33–2.45)	1.66 (1.22–2.26)
Severe	276	24/21.0	2.99 (2.00–4.46)	2.59 (1.73–3.87)	10/8.4	3.17 (1.70–5.92)	2.51 (1.34–4.69)	6/5.0	11.02 (4.86–25.00)	6.73 (2.95–15.35)	10/8.5	2.03 (1.09–3.78)	1.91 (1.02–3.56)
**Location of amputation**													
Shoulder/upper arm	28	1	1.38 (0.19–9.78)	1.25 (0.18–8.90)	0/0	-	-	0/0	-	-	1/9.6	2.30 (0.32–16.35)	2.22 (0.31–15.80)
Forearm	68	7	3.30 (1.57–6.92)	2.68 (1.28–5.63)	3/9.7	3.62 (1.17–11.26)	2.78 (0.89–8.65)	1/3.2	6.93 (0.97–49.58)	4.46 (0.62–32.09)	5/16.3	3.91 (1.62–9.40)	3.36 (1.40–8.10)
Wrist/hand	18,935	842	1.51 (1.40–1.64)	1.46 (1.34–1.58)	357/4.4	1.66 (1.47–1.89)	1.62 (1.42–1.83)	66/0.8	1.79 (1.34–2.41)	1.68 (1.24–2.26)	464/5.8	1.39 (1.25–1.55)	1.33 (1.19–1.48)
Hip/thigh	27	2	2.44 (0.61–9.78)	1.81 (0.45–7.26)	1/8.3	3.14 (0.44–22.34)	2.13 (0.30–15.13)	1/8.5	18.96 (2.65–135.63)	9.47 (1.31–68.43)	0/0	-	-
Lower leg	87	6	2.22 (1.00–4.96)	1.82 (0.82–4.05)	2/5.1	1.89 (0.47–7.56)	1.31 (0.33–5.27)	3/7.7	17.27 (5.50–54.25)	9.24 (2.92–29.18)	1/2.5	0.60 (0.09–4.27)	0.53 (0.08–3.77)
Ankle/foot	410	23	1.94 (1.28–2.92)	1.89 (1.25–2.85)	10/5.8	2.18 (1.17–4.07)	2.02 (1.08–3.76)	5/2.9	6.28 (2.57–15.35)	4.71 (1.92–11.54)	10/5.8	1.39 (0.75–2.59)	1.41 (0.76–2.64)

IR, incidence rate (numbers of cases per 1000 person–years). Model 1: Unadjusted. Model 2: Adjusted for age, sex, socioeconomic position (income level and place of residence), smoking, alcohol consumption, physical activity, body mass index, and comorbidities (hypertension, type 2 diabetes, and dyslipidemia).

## Data Availability

The data can be accessed upon request and approval of a proposal by the National Health Insurance System Database. Researchers whose proposed use of the data has been approved can access the deidentified participant data on the following website https://nhiss.nhis.co.kr/ (accessed on 2 July 2024).

## Supplementary Materials

Supplementary information is available for download at: https://www.mdpi.com/article/10.3390/healthcare12131362/s1, Table S1: Previous studies on the relationship between amputation and fracture; Table S2: Previous studies on the relationship between amputation and fracture; Table S3: Descriptions of severity degree in lower limb amputation.
