# Supplementary material for: Increased Risk of Fracture after Traumatic Amputation: A Nationwide Retrospective Cohort Study
Source: Healthcare (Basel). 2024 Jul 8;12(13):1362. doi: 10.3390/healthcare12131362 (PMC11241812; doi:10.3390/healthcare12131362)
Supplement: Supplementary file 1 [file healthcare-12-01362-s001.zip › healthcare-3010084-supplementary.pdf]

Supplement Table S1. Previous studies on the relationship between amputation and fracture

Author (year)	Country/Study design	Number of studies or subjects	Range of age or Mean age (years)	Cause of amputation (N or %)	Mean f/u period	Adjustment for covariates	Outcome
BMD as an outcome							
Finco et al. (2022)	-/review	27 studies<br><br>Transtibial: 10<br><br>Transfemoral: 7<br><br>Both: 9<br><br>Unclear: 2	NA	Traumatic: 15 studies<br><br>Dysvascular: 6<br><br>Cancer: 7<br><br>Congenital: 2	NA	NA	Reduced BMD<br><br>Increased muscle atrophy in amputees in amputated limb
Sherk et al. (2008)	USA/cross-sectional	Unilateral below-knee amputation (BKA): 7<br><br>Unilateral above-knee amputation (AKA): 7<br><br>Below-knee control (CBK): 7<br><br>Below-knee control (CAK): 6	23.3-63.9	Trauma: 11<br><br>diabetes: 1<br><br>circulation problems: 1<br><br>osteomyelitis: 1	NA	NA	**[DXA variables]**<br>- hip, trochanter BMD<br>BKA < CBK<br>- % diff between sides of hip sites<br>AKA > CAK<br>- % diff between limbs for hip BMD<br>BKA > CBK<br>- all hip BMD (BKA, AKA)

							Amputated. limb < intact limb<br>- % diff. in hip BMD between side<br>AKA (38–72%) > BKA (8-17%)<br>- Osteopenia for lumbar spine<br>AKA, BKA (36%) > CAK, CBK (8%)
Gait function or fall as an outcome							
Oliveira et al.<br>(2022)	-/scoping review	29 publications of 25 studies<br><br>252 with foot amputation<br><br>Toe<br>Great toe<br>Metatarsophalangeal<br>Ray<br>Transmetatarsal<br>More proximal~	26-75.5	Diabetes: 10 studies<br><br>Finger or thumb reconstruction: 5<br><br>Trauma: 4<br><br>Peripheral vascular disease: 3<br><br>Tumour: 1<br><br>Rheumatoid arthritis: 1<br><br>Congenital: 1<br><br>Frostbite: 1	NA	NA	**[Gait function]**<br>Toe: minor gait abnormalities<br><br>great toe: loss of push-off in a forward and lateral direction<br><br>Metatarsophalangeal: loss of stability and decreased gait speed<br><br>Ray: decreased gait speed and reduced lower

							extremity range of motion<br><br>Transmetatarsal, more proximal: abnormal gait, substantial loss of power generation across the ankle and impaired mobility.
Wong et al. (2021)	-/scoping review	21 articles<br><br>515 patients<br>Transtibial: 420 (81.6%)<br><br>Transfemoral: 79 (15.3%)<br><br>Bilateral: 15 (2.9%)	18-98 mean 53.2 (SD 22.1)	Trauma: 248 (48.2%)<br><br>Vascular: 144 (28.0%)<br><br>Medical/nonchronic: 45 (8.7%)<br><br>Other: 77 (15.0%)	NA	NA	**[Steps per day]**<br>All: 5087 (2998)<br>Transtibial: 5929 (3047)<br>Transfemoral: 3553 (2030)<br>Bilateral TFA: 1387<br>Vascular: 3339 (1889)<br>Traumatic: 4511 (1058)
Haleem et al. (2021)	United States/retrospective review	Amputee: 28<br><br>Control: 7787 (=standard hip fracture patients)	50-89 Mean 78.0 (SD 9.9)	Peripheral vascular: 13<br><br>Diabetes: 9<br><br>Rheumatoid arthritis of foot: 1	NA	NA	Fall at their home: 18<br><br>Mobility score on admission: amputee < control

							Able to return to pre-injury place: 70%<br><br>1-y mortality: insignificant Amputee (33.3%) vs Control (26.5%)
Major et al. (2020)	USA/cross-sectional study	Transradial: 8<br><br>Transhumeral: 3	Mean 50 (SD 18)	Congenital: 6<br><br>Acquired: 5	NA	NA	Upper limb loss: greater standing postural sway<br><br>Wearing prosthesis: improved weight-bearing symmetry, increased postural sway in medial-lateral direction
Major et al. (2019)	USA/cross-sectional study	Upper limb loss: 105<br><br>Through-elbow or proximal: 66<br><br>Below elbow: 32	18-82 Mean 43 (SD 17)	Congenital: 47<br><br>Cancer/tumor: 5<br><br>Accident/trauma: 42<br><br>Infection: 6	NA	BMI, physical activity, presence of LLL, ABC score, time since ULL, use of an upper limb prosthesis, physical capabilities	**[Fall]**<br>28.6%: 2 or more falls in past year<br><br>Reduced balance confidence<br><br>Reduced physical capabilities.

Miller et al. (2004)	Canada/prospective correlation study (two time point 2 years apart)	245<br><br>Below knee amputation (BKA): 68%<br><br>Above knee amputation (AKA): 32%	Mean 60.5 (SD 15.3)	Trauma: 56%	two time point 2 years apart	Baseline ABC score, years since amputation, comorbidity, no. of medications, problems with the good leg perceived health variable, fear of falling, baseline balance confidence score	**[Balance confidence]**<br>Mean ABC score < 80 (cut point)<br>: Baseline: 67.6<br>: Follow up: 68.0<br><br>Lower balance at follow-up<br>: Older, female, mobility device…
Miller et al. (2001)	Canada/cross-sectional	435<br><br>Below knee amputation (BKA): 73%<br><br>Above knee amputation (AKA) 27%	Mean 62 (SD 15.7)	Vascular: 53%<br><br>Nonvascular: 47%	NA	CES-D, amputation cause, level, back pain, joint pain, comorbidity, stump/prosthetic problems, BMI, year since amputation	**[Falling experience]**<br>Prevalence: 52.4%<br>AKA: OR 2.78 (1.71-4.51)<br>Back pain: OR 1.96 (1.08-3.54)<br>Joint pain: OR 1.67 (1.01-2.74)<br>Multiple stump and prosthesis problems: OR 3.09 (1.58-6.04)

							**[Fear of falling]** Prevalence: 49.2% Male: OR 0.35 (0.21-0.57)
Hermodsson et al. (1994)	Sweden/cross-sectional	Unilateral trans-tibial: 36<br><br>Healthy: 27	Vascular: 48-87 Mean 68.8 (SD 12.0)<br><br>Trauma: 48-82 Mean 63.9 (SD 10.0)	Vascular: 18<br><br>Trauma: 18	NA	Year since amputation, phantom sensation	Standing balance: vascular < trauma<br><br>Sway in the lateral direction : Vascular > healthy<br><br>Sway in the sagittal direction : trauma < vascular, healthy<br><br>Standing time in the one leg : vascular < trauma, healthy<br><br>Vascular <-> trauma should not be considered as an entity in test situations or

							rehabilitation programs.

BMD, Bone mineral density; DXA, central dual energy x-ray absorptiometry; TFA, Transfemoral amputation; BMI, Body mass index; LLL, Lower limb loss; ULL, Upper limb loss; ABC score, Activities-Specific Balance Confidence score; CES-D, Center for Epidemiologic Studies Depression Scale; OR, Odds ratio

Supplement Table S2. Definitions of severity degree in upper extremity amputation

Grade		Definitions
Level	Number	
1	1	Amputation above the wrist joint of both arms
2	1	Amputation of both thumbs above IP joint and all 2nd to 5th fingers above PIP joint
	2	Amputation above the elbow joint of one arm
3	1	Amputation of both thumbs above IP joint and 2nd finger above PIP joint
	2	Amputation of one thumb above IP joint and all 2nd to 5th fingers above PIP joint
4	1	Amputation of both thumbs above IP joint
	2	Amputation of one thumb above IP joint and 2nd finger above PIP joint
	3	Amputation of one thumb above IP joint and other two fingers above PIP joint
5	1	Amputation of one thumb above IP joint and the other one finger above PIP joint
	2	Amputation of one thumb above MCP joint
	3	Amputation of three fingers above PIP joint including 2nd finger
6	1	Amputation of one thumb above IP joint
	2	Amputation of two fingers above PIP joint including 2nd finger
	3	Amputation of all 3rd to 5th finger of one hand above PIP joint

IP, Interphalangeal; PIP, proximal interphalangeal; MCP, metacarpophalangeal

Supplement Table S3. Definitions of severity degree in lower extremity amputation

Grade		Definitions
Level	Number	
1	2	Amputation of both legs above the knee joint
2	3	Amputation of both legs above the ankle joint
3	3	Amputation of both legs above the transverse tarsal joint (Chopart joint)
	4	Amputation of one leg above the knee joint
4	4	Amputation of both legs above the tarsometatarsal joint (Lisfranc joint)
	5	Amputation of one leg above the ankle joint
5	4	Amputation of both big toes above the interphalangeal joint and 2nd to 5th toes above the proximal interphalangeal joint
	5	Amputation of one leg above the transverse tarsal joint (Chopart joint)
6	4	Amputation of one leg above the tarsometatarsal joint (Lisfranc joint)
